# Can Illness Perceptions Predict Lower Heart Rate Variability following Acute Myocardial Infarction?

**DOI:** 10.3389/fpsyg.2016.01801

**Published:** 2016-11-18

**Authors:** Mary Princip, Marco Scholz, Rebecca E. Meister-Langraf, Jürgen Barth, Ulrich Schnyder, Hansjörg Znoj, Jean-Paul Schmid, Julian F. Thayer, Roland von Känel

**Affiliations:** ^1^Department of Neurology, Inselspital, Bern University Hospital, and University of BernBern, Switzerland; ^2^Psychosomatic Research Group, Department of Clinical Research, University of BernBern, Switzerland; ^3^Department of Cardiology, Inselspital, Bern University HospitalBern, Switzerland; ^4^Clienia Schlössli AG, Private Psychiatric and Psychotherapy ClinicOetwil am See, Switzerland; ^5^Institute for Complementary and Integrative Medicine, University Hospital ZurichZurich, Switzerland; ^6^Department of Psychiatry and Psychotherapy, University Hospital Zurich, University of ZurichSwitzerland; ^7^Division of Clinical Psychology and Psychotherapy, Institute of Psychology, University of BernBern, Switzerland; ^8^Cardiology Clinic, Tiefenauspital, Bern University HospitalBern, Switzerland; ^9^Department of Psychology, Ohio State University ColumbusColumbus, OH, USA; ^10^Department of Psychosomatic Medicine, Clinic BarmelweidBarmelweid, Switzerland

**Keywords:** heart rate variability, illness perceptions, myocardial infarction, longitudinal study, psychological stress

## Abstract

**Objective:** Decreased heart rate variability (HRV) has been reported to be a predictor of mortality after myocardial infarction (MI). Patients' beliefs and perceptions concerning their illness may play a role in decreased HRV. This study investigated if illness perceptions predict HRV at 3 months following acute MI.

**Methods:** 130 patients referred to a tertiary cardiology center, were examined within 48 h and 3 months following acute MI. At admission, patients' cognitive representations of their MI were assessed using the German version of the self-rated Brief Illness Perception Questionnaire (Brief IPQ). At admission and after 3 months (follow-up), frequency and time domain measures of HRV were obtained from 5-min electrocardiogram (ECG) recordings during stable supine resting.

**Results:** Linear hierarchical regression showed that the Brief IPQ dimensions timeline (β coefficient = 0.29; *p* = 0.044), personal control (β = 0.47; *p* = 0.008) and illness understanding (β = 0.43; p = 0.014) were significant predictors of HRV, adjusted for age, gender, baseline HRV, diabetes, beta-blockers, left ventricular ejection fraction (LVEF), attendance of cardiac rehabilitation, and depressive symptoms.

**Conclusions:** As patients' negative perceptions of their illness are associated with lower HRV following acute MI, a brief illness perception questionnaire may help to identify patients who might benefit from a specific illness perceptions intervention.

## Introduction

Myocardial infarction (MI) is the leading cause of morbidity and mortality in developed countries (Murray and Lopez, 1997). Heart rate variability (HRV) plays an important role in the risk of morbidity and mortality of cardiovascular disease (Darwin, 1998). HRV is a non-invasive tool to measure the cyclic variations of beat-to-beat (RR) intervals reflecting cardiac autonomic modulation (Akselrod et al., 1981). In one of the first studies investigating the relationship between indices of HRV and mortality, Kleiger et al. (1987) showed that decreased HRV was a significant and independent predictor of mortality in post-MI patients. Further studies have since documented adverse prognostic implications of reduced HRV in MI patients (Cripps et al., 1991; Tsuji et al., 1996; Boskovic et al., 2014; Song et al., 2014). Psychosocial factors, such as stressful life events (Pieper et al., 2010; van Ockenburg et al., 2015), general stress (Kang et al., 2004; Brosschot et al., 2006; Chandola et al., 2010; Chida and Steptoe, 2010), hostility (Virtanen et al., 2003; Chida and Steptoe, 2009), depression (Stein et al., 2000; Gehi et al., 2005; Ha et al., 2015; Sgoifo et al., 2015) and anxiety (Friedman and Thayer, 1998; Alvares et al., 2013) have all been found to be associated with lowered HRV. In addition, individuals with stronger emotion regulation (Thayer et al., 2009; Patron et al., 2014; Gillie et al., 2015) and adaptive coping strategies have been shown to have higher levels of HRV (Appelhans and Luecken, 2008; Thayer and Lane, 2009). Moreover, low cardiac vagal tone, as reflected in the square root of the mean of the squared differences between adjacent normal RR intervals (rMSSD), is associated with poor self-regulation and lack of behavioral flexibility (Porges, 1992). Psychosocial factors in their own right have also been emerging as risk factors of incident MI and carry a poor prognosis of recovery from MI (Carney et al., 2005; Thayer et al., 2010).

Patients' cognitive representations and perceptions of their illness play an important role in different aspects of recovery from MI (Petrie and Weinman, 2006; Broadbent et al., 2006a). The construct of illness perceptions is based on the self-regulatory model (Leventhal, 1984; Leventhal, and Steele, 1984). This model proposes that patients are active problem solvers who cope with their illness threats by personal cognition (Leventhal and Steele, 1984). Patients' negative beliefs and perceptions about their illness are associated with pre-hospital delay (decision-time) (Vidotto et al., 2013) lower adherence to therapy (French et al., 2005), delayed return to work (Foxwell et al., 2013), impaired functioning (Petrie et al., 1996), and emotional distress (Scharloo et al., 1998; Watkins et al., 2000). In cardiac patients, illness perceptions, including loss of perceived control, negative emotional responses and greater consequences have variously been shown to be associated with psychological distress, such as anxiety and depressive symptoms (Sherry et al., 2005). Moreover, a range of psychosocial factors, including depression (Stein et al., 2000; Gehi et al., 2005; Ha et al., 2015; Sgoifo et al., 2015) and anxiety (Friedman and Thayer, 1998; Alvares et al., 2013), but also stressful life events (Pieper et al., 2010; van Ockenburg et al., 2015), general stress (Kang et al., 2004; Brosschot et al., 2006; Chandola et al., 2010; Chida and Steptoe, 2010) and hostility have all been found to be associated with lowered HRV. In addition, negative illness perceptions are associated with maladaptive health behaviors, such as lack in physical activity, which in turn might contribute to lower HRV. Therefore, psychological distress and maladaptive coping with the cardiac disease might be mechanisms that contribute to the link between negative illness perceptions with HRV over time. While previous studies have explored the effect of illness perceptions on physical activity in patients suffering from cardiac illness (Petrie et al., 1996; Murray and Lopez, 1997; Petrie and Weinman, 2006), the relationship between illness perceptions and HRV has not been addressed so far, particularly in a longitudinal setting. Moreover, there are several studies that demonstrate the predictive value of HRV on health outcomes (Kleiger et al., 1987; Darwin, 1998). Through an evaluation of illness perception in relation to HRV over a longer time, patients with an increased cardiovascular risk could be identified and possibly treated to improve cardiovascular outcome. Therefore, the purpose of this exploratory study was to examine the association between HRV and illness perceptions in patients over a 3-month period after MI.

## Methods

### Study participants and design

One hundred and thirty consecutive acute MI patients admitted to the Coronary Care Unit (CCU) of a tertiary university center were enrolled in the Myocardial Stress Prevention Intervention (MI-SPRINT) randomized controlled trial aimed at reducing the incidence of posttraumatic stress by an early behavioral intervention. The research protocol was approved by the ethics committee of the State of Bern, Switzerland. The study protocol with detailed inclusion and exclusion criteria has been described elsewhere (Meister et al., 2013). In brief, eligible patients were 18 years and older, spoke German and had a positive distress screening at the time of hospital admission; i.e., they scored at least 5 for chest pain plus at least 5 for fear of dying and/or helplessness on numeric rating scales ranging from 0 (e.g., *no pain at all*) to 10 (e.g., *unbearable pain*). After informed consent had been obtained, patient's illness perceptions and HRV were assessed within 48 h after MI onset. At 3 months after enrolment in the study, all study participants were invited for a follow-up assessment.

### Assessment of medical status

Patients' medical status was obtained with a structured medical history. Data on diabetes, hypertension, left ventricular ejection fraction (LVEF) and medical baseline (medication intake) at admission and at the 3-month of follow-up were assessed.

### Psychometric assessment

#### Illness perceptions

Patients' cognitive representations were assessed with the German version of the self-rated Brief Illness Perception Questionnaire (Brief IPQ) (www.uib.no/ipq/html/b-ipq.html) (Broadbent et al., 2006b). The Brief IPQ consists of 9 questions and represents different dimensions of the illness. These dimensions are: 1 *consequences* (How much does your illness affect your life?), 2 *timeline* (How long do you think your illness will continue?), 3 *personal control* (How much control do you feel you have over your illness?), and 4 *treatment control* (How much do you think your treatment can help your illness?) assess cognitive representations. Emotional representations are measured by dimension 6 *illness concern* (How concerned are you about your illness?) and 8 *emotions* (How much does your illness affect you emotionally?). Dimension 5 *identity* (How much do you experience symptoms from your illness?) represents individual intensity of symptoms, while dimension 7 *coherence* (How well do you feel you understand your illness?) assesses illness comprehension. Each dimension, except the causal item, is rated using a 0 to 10 response scale, with 10 indicating highest intensity. Assessment of the causal representation (item 9) is by an open-ended response item, which asks patients to list the three most important self-assumed causes of their MI. Brief IPQ showed moderate overall test-retest reliability (Leysen et al., 2015) and good concurrent and predictive validity (Broadbent et al., 2015).

#### Depressive symptoms

The Beck Depression Inventory (BDI-II) (Ahrari et al., 2013) was used to measure depressive symptoms as an important factor potentially affecting HRV. In order to reduce the possible overlap between depressive and MI-associated physical symptoms, we only applied the cognitive/affective symptom subscale. The validated German version of the BDI-II (Kuhner et al., 2007) is a self-report questionnaire and comprises 13 items to be rated on a 4-point Likert scale. Total scores range from 0 to 39, with 0 to 3 representing no depressive symptoms, 4–6 mild and 7–9 moderate depressive symptoms. Scores of at least 10 indicate clinically relevant depressive symptoms. The reliability of the cognitive symptom subscale of the BDI-II was acceptable in our sample (Cronbach's α = 0.71).

#### Analysis of HRV

Continuous electrocardiogram (ECG) recordings were performed in each patient for 5-min periods during stable supine resting (Agelink et al., 2001) using a Finometer device (TNO Biomedical Instrumentation, Amsterdam, The Netherlands). The Finometer records the beat-to-beat finger pulse contour and assesses short-term changes of blood pressure and its variability. All cardiovascular variables were stored digitally in result files on a hard disk. The Beatscope 1.1a software program integrates the subject's gender, age, BMI and is used to obtain HRV parameters (Wesseling et al., 1986). Using Beatscope 1.1a, inter-beat intervals (IBI in milliseconds) were exported to a single text file.

In a further step, IBIs were exported to an Excel sheet, where clear artifacts (IBI > 1800 ms; ≤ 300 ms) were identified and manually removed. Time and frequency domain measures of the heart period power spectrum were analyzed and performed using the Kubios HRV analysis package 2.2 (Biosignal Analysis and Medical Imaging Group (BSAMIG); http://kubios.uef.fi/). We corrected artifacts with Kubios HRV by applying cubic spline interpolation to replace missing IBIs. We obtained spectral estimates of HRV power (in milliseconds squared per hertz). In the frequency domain methods, the heart rate (HR) time series is partitioning the HR variance into spectral components and quantifying their power (Wesseling et al., 1986). The power spectrum was calculated by using the autoregressive model (Thayer et al., 2002) to obtain total power of HRV and its main components: high frequency (HF, 0.15–0.4 Hz) and low frequency (LF, 0.04–0.15 Hz). Total power of HRV is an estimate of the global activity of the autonomic nervous system. The HF power is considered an indicator of cardiac parasympathetic activity. The LF component of HRV is mediated by both sympathetic and parasympathetic activities. The LF/HF ratio of HRV has been proposed an index of cardiac sympathovagal activity balance (1996; Thayer et al., 2002). The rMSSD was computed from time domain measures. Both indices, the HF power and rMSSD, are exclusively mediated by the vagus nerve (1996). Mean HR were analyzed to measure autonomic balance. Total power, HF power, LF power and rMSSD were logarithmically transformed (base 10) to achieve a normal distribution (Thayer et al., 2002).

### Data analysis

Statistical analysis was performed using SPSS software version 22.0 (Chicago, Illinois, USA). Results of statistical tests with *p* < 0.050 (two-tailed) were considered significant. Results were expressed as mean ± standard error of the mean. Data were verified for normal distribution using the Kolmogorov-Smirnov test. Group differences were calculated using Student's *t*-test for normally distributed variables and Mann-Whitney *U*-test for non-parametric variables. Bivariate correlations between two variables were estimated with Spearman coefficients. Hierarchical linear regression modeling explored the predictive value of illness perceptions for HRV indices with adjustment for age, gender, baseline HRV, diabetes, beta-blocker use, LVEF, cognitive depressive symptoms and attendance of cardiac rehabilitation. Data were checked for multicollinearity by Durban Watson statistics and linearity of residuals was verified by means of scatter plots.

## Results

### Patient characteristics

Table 1 shows the patient characteristics at the time of MI. The mean age of all participants was 60.3 ± 10.4 years. All participants were of European origin (*N* = 130), with the majority being male (81.4%). Nineteen participants were lost to the 3-month follow-up for different reasons, such as “no interest to participate anymore,” death or loss of contact information. Therefore, from the original cohort of 130 persons, 111 were available for the 3-month follow-up investigation.

**Table 1:** **Characteristics of study subjects (mean ± SD or %; N = 130)**.

**Characteristics**	**Mean ± SD**	**%**
Age	60.3±10.4	
Male gender		81.4
**MARITAL STATUS**		
Married		63.8
Divorced		16.9
Widowed		5.4
Single		13.8
**WORKING STATUS**		
Full time		43.8
Part time		12.3
Retired/unemployment		43.8
**HIGHEST LEVEL OF EDUCATION**		
Lower than apprenticeship		11.8
Apprenticeship		70.1
High school		3.9
University		14.2
BMI, kg/m^2^	27.8±4.9	
Systolic blood pressure (mmHg)	139.5±28.7	
Diastolic blood pressure (mmHg)	81.7±14.9	
Diabetes		18.7
Hypertension		56.5
Hypercholesterolaemia		55.3
Beta-blockers		20.6
Antidepressants		8.1
Benzodiazepines		2.2
Smokers		42.3
Physical activity		47.7
Attendance at cardiac rehabilitation		
program		66.2
LVEF	48.4±12.5	
Previous myocardial infarction		11.5
Total duration of stay in hospital	3.7±2.9	

*SD, standard deviation; BMI, body mass index; LVEF, left ventricular ejection fraction*.

### Associations and differences between HRV and covariates at admission

Heart rate variability (HRV) indices did not significantly differ between men and women. Older patients showed significantly decreased HRV compared to younger patients in terms of total power (*r* = −0.19; *p* = 0.037) at admission. Patients with diabetes showed significantly lower total power (3.1 ± 0.53 vs. 2.7 ± 0.44, *p* = 0.014) at admission and higher HR (69.4 ± 9.6 vs. 62.4 ± 9.2, *p* = 0.024) at the 3-month follow-up compared to patients without diabetes. There was a significant difference neither between normotensive and hypertensive patients nor between smokers and non-smokers at admission and at the 3-month follow-up in HRV indices. Patients on beta-blockers showed significantly lower HR at admission (65.3 ± 10.6 vs. 72.3 ± 12.2 vs. *p* = 0.039) and lower LF power (2.3 ±0.55 vs. 2.8 ± 0.28, *p* = 0.001) at 3 months follow-up. LVEF was negatively correlated with HR at admission (*r* = −0.20; *p* = 0.034). HRV indices of patients who attended a cardiac rehabilitation program did not differ from those of patients who did not. There were no significant correlations between depressive symptoms (BDI-II) and HRV indices across time points.

### Bivariate associations of brief IPQ dimensions with HRV

**Figure 1:**
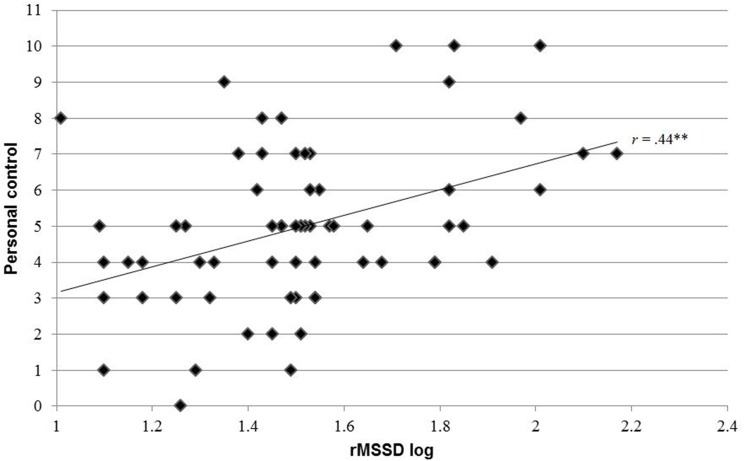
Pearson correlation between personal control at admission and rMSSD 3 months post-MI; rMSSD, square root of the mean of the squared differences between adjacent normal RR intervals; Ml, myocardial infarction; ^**^p < 0.001.

At admission, HRV indices were significantly correlated with the dimensions *timeline* (total power: *r* = 0.24; *p* = 0.024; LF power: *r* = 0.25; *p* = 0.028; HF power: *r* = −0.35; *p* = 0.002; LF/HF ratio: *r* = −0.29; *p* = 0.007; rMSSD: *r* = 0.38; *p* = 0.014), *treatment control* (LF/HF ratio: *r* = −0.21; *p* = 0.045) and *emotional representation* (HF power: *r* = 0.24; *p* = 0.034). With the exception of mean resting HR, all HRV indices showed significant correlations with Brief IPQ dimension *timeline*. At 3 months follow-up, *timeline* (HR: *r* = 0.24; *p* = 0.031; rMSSD: *r* = −0.34; *p* = 0.037), *personal control* (rMSSD: *r* = 0.44; *p* < 0.001), *identity* (LF/HF ratio: *r* = −0.29; *p* = 0.013) and *illness coherence* (HF: *r* = 0.43, *p* = 0.008) showed significant associations with HRV indices. Figure 1 shows the association of vagally-mediated HRV, expressed in rMSSD, and the assumption of higher *personal control* over the illness.

### Mean HRV differences within 3 months

There were no significant changes over time in the HRV indices (total power, HF power, LF power, LF/HF power, rMSSD). Mean HR had significantly decreased from admission to the 3-month follow-up (71.66 ± 12.69 vs. 62.12 ± 9.18, *p* < 0.001).

### Comparison of different predictors for HRV at 3-months follow-up

Tables 2, 3 show the results of the hierarchical linear regression analysis with four models (1a–1d), in which four blocks of covariates were linked to HRV indices (HF power and resting HR). Beta-blockers, LVEF and depressive symptoms did not emerge as significant predictors for HF power and HR in any model, whereas gender and attendance of rehabilitation program showed significance in the adjusted final model (1d). Age and baseline HR emerged as significant predictors of resting HR in the final model (2d).

**Table 2:** **Illness coherence and illness control as predictors of HF Power at 3 months after acute MI**.

**Statistics of entire model**	**Entered variables**	***B***	**S.E**.	**β**	***p***	***r^2^***
*Model 1a*	Age	−0.002	0.007	−0.050	0.74	0.230
[*F*_(2, 111)_ = 3.678, *p =* 0.021, adjusted *R*^2^ = 0.167]	Gender	0.482	0.161	−0.443	0.005	0.186
	HF baseline	0.090	0.123	0.112	0.47	0.011
*Model 1b*	Age	−0.001	0.007	−0.023	0.88	0.001
[*F*_(5, 111)_ = 1.983, *p* = 0.096, adjusted *R*^2^ = 0.128]	Gender	0.418	0.179	0.384	0.026	0.118
	HF baseline	0.101	0.127	0.124	0.44	0.013
	Diabetes	−0.141	0.249	−0.090	0.57	0.007
	Beta blockers	0.113	0.196	0.091	0.57	0.007
	LVEF	0.004	0.007	0.091	0.61	0.005
*Model 1c*	Age	−0.003	0.007	−0.059	0.70	0.003
[*F*_(7, 111)_ = 2.324, *p* = 0.043, adjusted *R*^2^ = 0.209]	Gender	0.486	0.173	0.446	0.009	0.155
	HF baseline	0.154	0.126	0.191	0.23	0.029
	Diabetes	−0.160	0.238	−0.102	0.51	0.009
	Beta blockers	−0.063	0.215	0.029	0.77	0.001
	LVEF	0.002	0.007	0.132	0.79	0.001
	BDI	0.023	0.025	0.152	0.37	0.016
	Rehabilitation	−0.465	0.199	−0.394	0.026	0.108
*Model 1d*	Age	−0.006	0.007	−0.133	0.41	0.011
[*F*_(10, 111)_ = 2.989, *p* = 0.009, adjusted *R*^2^ = 0.354]	Gender	0.406	0.162	0.373	0.018	0.101
	HF baseline	0.201	0.115	0.249	0.091	0.049
	Diabetes	−0.435	0.235	−0.277	0.074	0.055
	Beta blockers	0.116	0.204	0.093	0.58	0.005
	LVEF	−0.006	0.007	−0.165	0.35	0.014
	BDI	0.020	0.024	0.133	0.40	0.011
	Rehabilitation	−0.301	0.213	−0.286	0.049	0.083
	Timeline	−0.029	0.025	−0.197	0.25	0.022
	Personal control	0.103	0.038	0.443	0.011	0.118
	Coherence	0.079	0.031	0.426	0.014	0.109

*HF, high frequency; MI, myocardial infarction; S.E., standard error; LVEF, left ventricular ejection fraction; BDI, Beck Depression Inventory*.

**Table 3:** **Timeline as predictor of HR at 3 months after acute MI**.

**Statistics of entire model**	**Entered variables**	***B***	**S.E**.	**β**	***p***	***r^2^***
*Model 2a*	Age	−0.155	0.124	−0.149	0.22	0.022
[*F*_(2, 111)_ = 11.196, *p* < 0.001, adjusted *R*^2^ = 0.433]	Gender	−4.510	2.907	−0.187	0.13	0.034
	HR baseline	−0.500	0.099	−0.697	0.001	0.360
*Model 2b*	Age	−0.142	0.120	−0.137	0.24	0.018
[*F*_(5, 111)_ = 7.331, *p* < 0.001, adjusted *R*^2^ = 0.487]	Gender	−5.310	3.101	−0.220	0.096	0.037
	HR baseline	−0.513	0.101	−0.624	< 0.001	0.328
	Diabetes	9.458	4.300	0.271	0.035	0.062
	Beta blockers	−4.333	3.326	−0.157	0.20	0.021
	LVEF	0.214	0.121	0.246	0.085	0.040
*Model 2c*	Age	−0.135	0.119	−0.130	0.26	0.015
[*F*_(7, 111)_ = 6.301, *p* < 0.001, adjusted *R*^2^ = 0.515]	Gender	−6.197	3.136	−0.257	0.057	0.047
	HR baseline	−0.483	0.102	−0.587	< 0.001	0.271
	Diabetes	9.692	4.186	0.277	0.028	0.064
	Beta blockers	−0.504	3.777	−0.018	0.90	0.001
	LVEF	0.227	0.120	0.260	0.068	0.043
	BDI	−0.824	0.440	−0.244	0.071	0.042
	Rehabilitation	4.784	3.504	0.183	0.18	0.022
*Model 2d*	Age	−0.234	0.135	−0.226	0.039	0.033
[*F*_(10, 111)_ = 5.574, *p* < 0.001, adjusted *R*^2^ = 0.557]	Gender	−5.389	3.170	−0.224	0.10	0.032
	HR baseline	−0.508	0.102	−0.617	< 0.001	0.275
	Diabetes	6.647	4.383	0.190	0.14	0.025
	Beta blockers	−0.626	3.705	−0.023	0.87	0.001
	LVEF	0.039	0.129	0.107	0.48	0.005
	BDI	−0.679	0.431	−0.201	0.13	0.027
	Rehabilitation	7.142	3.995	0.273	0.084	0.035
	Timeline	0.964	0.458	0.292	0.044	0.049
	Personal control	0.014	0.704	0.003	0.98	0.001
	Coherence	0.705	0.562	0.170	0.22	0.017

*HR, heart rate; MI, myocardial infarction; S.E., standard error; LVEF, left ventricular ejection fraction; BDI, Beck Depression Inventory*.

Regarding illness perceptions, HF power at 3 months follow-up was significantly predicted by the dimension *personal control* and the dimension *illness coherence* in the final model (1d). After taking into account all covariates, the illness perceptions dimensions *personal control, illness coherence* and *timeline* explained 24% of the total variance. As illustrated in Table 3, *timeline* emerged as an independent predictor of resting HR, explaining 5% of the total variance.

Similar results were obtained with rMSSD as the dependent variable and the dimension *personal control* as an independent variable adjusted for age, gender, baseline rMSSD, diabetes, LVEF, attendance at cardiac rehabilitation and depressive symptoms. Specifically, baseline rMSSD (standardized β coefficient = −0.37; *p* = 0.033) emerged as a significant predictor in the final model, explaining 10% of the total variance. None of the other covariates made a significant contribution to the predictive power in the final model.

Regarding illness perceptions, *personal control* (standardized β coefficient = 0.47; *p* = 0.008) emerged as a significant predictor in the final model, explaining 17% of the total variance.

Beta-blockers emerged as an independent predictor for LF power (standardized β coefficient = −0.40; *p* = 0.004) after taking into account all covariates.

Timeline, personal control and illness understanding did not show independent associations with LF power, LF/HF ratio and total power. Further, none of the other illness perception dimensions (consequences, treatment control, identity, illness concern and emotional representation) showed a significant association with all HRV indices in our regression model.

The adjusted R^2^ value for the final models was 0.35 for the HF power, 0.55 for resting HR and 0.27 for rMSSD, indicating that roughly 40% of the variance in the selected HRV indices was accounted for by the entire set of covariates.

## Discussion

We found significant associations between Brief IPQ variables assessed within 48 h of an acute MI and HRV indices assessed at hospital admission as well as 3 months later. Five out of eight general illness perception factors showed a significant association with HRV indices. The results suggest that HRV may be affected by patients' understanding of the cardiac event, and their control over the MI and its emotional impact on their lives.

The main finding of this study was that personal control over the illness and coherence about the illness were significant predictors of vagally-mediated HRV at 3 months after acute MI. Moreover, patients' idea about how long the MI would last (dimension *timeline*) predicted resting HR at 3 months after MI. Interestingly, only purely vagally-mediated HRV indices (i.e., HF power and rMSSD) and resting HR were predicted from illness perception dimensions, whereas the other HRV indices (i.e., LF, total power, LF/HF) were not. It has to be pointed out that the dimension *timeline* is less meaningful for MI than the other dimensions, because it is a one-time event with irreversible damage.

To our knowledge, these results are novel and support the growing body of evidence that illness perceptions play a key role in recovery from MI. Research has shown that a brief illness perceptions intervention can change MI-related negative illness beliefs, and it also reduces illness anxiety in spouses of post-MI patients (Appelhans and Luecken, 2008; Thayer and Lane, 2009). Therefore, implementing prevention strategies, such as cognitive interventions targeting understanding of illness after acute MI might improve general functioning as well as HRV. Our results also support the notion that HRV indices, specifically those reflecting vagal function, are associated with emotional states (Brosschot et al., 2006) and maladaptive coping strategies (Appelhans and Luecken, 2008; Thayer and Lane, 2009).

Apart from understanding the illness and perceived control over the illness, gender, attendance of rehabilitation program and age also had a significant effect on HRV values measures at the 3-month follow-up. This is in line with previous research findings which showed that LF power was reported to be significantly lower in healthy women compared to healthy men (Ramaekers et al., 1998). Further, a significant increase of HF power and LF power in MI patients was found after 8 weeks of cardiac rehabilitation (Sandercock et al., 2007). Moreover, older patients showed reduced HRV in total power and both LF and HF power compared to younger patients (Zhang, 2007).

Additionally, there was a significant recovery of autonomic balance reflected by a significant decrease in HR between admission and 3 months of follow-up. Other HRV indices showed no significant changes over 3 months following acute MI. These results are relatively consistent with the findings of Vaage-Nilsen et al. (2001) who showed that HRV measured during the day did not change over one-and-a-half years following acute MI, but was significantly reduced compared with healthy men. An explanation for the significant decrease of resting HR within 3 months could be that cardiac rehabilitation was an independent predictor of resting HR in our study sample. These findings highlight the potential of rehabilitation programs to improve recovery after a cardiac event.

## Limitations

Several methodological limitations of our study need to be mentioned. The Brief IPQ was assessed at admission only. Therefore, an evaluation of possible changes over time in illness perceptions on HRV was not possible. However, this study was one of the first to assess illness perceptions very shortly after MI. Severe depression was an exclusion criterion for participation in the study. Consequently, the variance in the severity of depressive symptoms at admission was rather small and thus might help explain why depressive symptoms did not predict HRV measures. Since the MI-SPRINT parent study is an intervention study, it is also possible that HRV indices were moderated by the two different interventions performed at admission. As the trial is still running, we could not break the blinding. The external validity of our findings is limited because only patients referred to a tertiary center were included. The limited sample size must also be considered when interpreting the HRV findings. Even if the study design controlled for different covariates, HRV might be influenced by other processes after the traumatic event, such as coping strategies, cardiovascular fitness or personality traits. Thus, although the study applied a prospective design, causal inferences cannot be drawn from our results.

## Conclusions

This study highlights the importance of illness understanding and control over the illness in MI patients for vagal modulation of the heart. Therefore, identifying patients with lower internal locus of control and lack of illness understanding might help to select patients at high risk for negative health outcome and offer them support in coping with the cardiac disease. Implementing psychoeducation and psychotherapeutic interventions in MI patients, targeting illness perceptions, has been shown to be predictive for lower anxiety and greater increases in exercise (Broadbent et al., 2009) and might therefore increase HRV and thus possibly reduce the risk of recurrent cardiac events. Such interventions and their potential beneficial effect on outcomes ought to be tested in future trials. A more detailed examination of the coping mechanism such as resilience or self-efficacy underlying the promoting effect of illness perception on HRV needs to be addressed in future studies.

Taken together, the results of this study show that illness perceptions independently predict vagally-mediated HRV indices assessed at 3 months after acute MI. Thus, the findings might help explain the poor cardiovascular prognosis in post-MI patients as related to decreased HRV.

A brief illness perception questionnaire used in clinical routine might help assess the need for correcting maladaptive beliefs, and implementation of psychotherapeutic interventions may possibly improve HRV further downstream.

This study is a first step toward a better understanding of the changes in HRV in a longitudinal setting and the role of illness perception in the context of acute MI.

## Author contributions

RvK, JS, US, JB, and HZ contributed to the development of the study design. US, HZ, JB, RvK, RM, and MP contributed to the development of the verum and control intervention. MS, RM, and MP contributed to the recruitement of the patients in hospital. MP and MS wrote the first draft of the manuscript. All authors critically revised and approved the final manuscript.

## Trial registration number

NCT01781247 (https://clinicaltrials.gov/ct2/show/NCT01781247).

### Conflict of interest statement

The authors declare that the research was conducted in the absence of any commercial or financial relationships that could be construed as a potential conflict of interest.

## Abbreviations

BDI: Beck Depression Inventory
Brief IPQ: Brief Illness Perception Questionnaire
CCU: Coronary Care Unit
ECG: electrocardiogram
FU: follow-up
HR: heart rate
HRV: heart rate variability
HF: high frequency
IBI: inter-beat intervals
LF: low frequency
LVEF: left ventricular ejection fraction
MI: myocardial infarction
MI-SPRINT: Myocardial Stress Prevention Intervention
rMSSDm: square root of the mean of the squared differences between adjacent normal RR intervals.
